# Addition of a surfactant to water increases the acaricidal activity of extracts of some plant species used to control ticks by Zimbabwean smallholder farmers

**DOI:** 10.1186/s12917-019-2078-3

**Published:** 2019-11-09

**Authors:** Emmanuel T. Nyahangare, Brighton M. Mvumi, Lyndy J. McGaw, Jacobus N. Eloff

**Affiliations:** 1 Department of Animal Science, Faculty of Agriculture, University of Zimbabwe, P O Box MP167, Mt Pleasant, Harare, Zimbabwe; 2 Phytomedicine Programme, Faculty of Veterinary Science, University of Pretoria, Private Bag X04, Onderstepoort, Pretoria, 0110 South Africa; 3 Department of Soil Science & Agricultural Engineering, Faculty of Agriculture, University of Zimbabwe, P O Box MP167, Mt Pleasant, Harare, Zimbabwe

**Keywords:** Water extracts, Acetone, Solvent-solvent fractionation, Cattle tick, SLIT bioassay, Tick larvae mortality, Biological activity

## Abstract

**Background:**

Many studies have revealed that bioactive compounds for different indications are not extracted from plants with water, the only extractant practically available to rural communities. We compared the acaricidal activity of acetone extracts of 13 species used traditionally to protect cattle against ticks. We also investigated if the extraction of biologically active compounds against *Rhipicephalus (Boophilus) decoloratus* ticks could be enhanced by adding a liquid soap that is locally available to smallholder farmers.

**Methods:**

A total of 13 plant species selected based on reported traditional use in Zimbabwe, were dried and finely ground before extraction with water, or water plus a surfactant, or acetone. The adapted Shaw Larval Immersion Test (SLIT) method was used to determine the activity of acetone and crude water extracts with or without liquid soap against the tick larvae. The activity of four fractions of crude acetone extracts (extracted using solvents of different polarity), of the most active plant species, Maerua edulis (tuber and leaf) was also compared to identify the most active fraction.

**Results:**

Aqueous plant extracts were not toxic to ticks, but the addition of 1% liquid soap as a surfactant increased mortality of the *R. (B) decoloratus* larvae significantly. With the *Maerua edulis* tuber extract, the efficacy of the 1% liquid soap was comparable to that of the amitraz based commercial synthetic acaricide. The use of acetone as an extractant, also increased the mortality of the tick larvae in all the plant species. With *M. edulis* (tuber and leaf), *Monadenium lugardae* and *Kleinia* sp. acetone extracts, the activity was comparable to that of the positive control (a commercially available amitraz-based synthetic acaricide). The non-polar fractions of the acetone extract of leaf and tuber of *M. edulis* caused up to 100% mortality. This indicates that non-polar to intermediate polarity compounds are responsible for the acaricidal activity.

**Conclusion:**

Organic solvents such as acetone extracted active compounds but water did not. By adding commonly available dishwashing soap to water active compounds were extracted leading to a high acaricidal activity of the plant extracts. In some cases, it was as active as non-polar extracts and a synthetic commercial acaricide (positive control). This approach makes it possible for the smallholder farmers and traditional healers to extract biologically active compounds from plants by using water.

## Background

Ticks cause major problems to optimal livestock productivity and may also cause human diseases especially in tropical parts of the world by the transfer of pathogens [1]. Ticks also affect animals through tick-worry and hide damage [2]. The global cost of managing and controlling ticks and tick-borne diseases are estimated to be several billions of United States dollars annually [3]. For many years, chemical control using synthetic acaricides has been the preferred control strategy but there are now several legitimate issues against their extensive use. Synthetic acaricides are expensive, active environmental pollutants, have been found as residues in animal products and their continual use has led to development of tick resistance to most acaricides on the market [4]. These are some of the issues that researchers are trying to address by investigating alternative or complementary products that farmers can use.

Many scientists are investigating the use of botanical pesticides as effective, safe and environmentally benign products against ticks [5]. Historically, plants played pivotal roles in agricultural pest management, but the advent of synthetic compounds had reduced this role. There is, consequently, a need to identify and verify activity of plants that are useful from the many plant species in the world. This is already a budding industry with potential to alleviate the challenges of tick control using synthetic products. There are several recent review papers that address various aspects such as: which plants have been used traditionally to combat ticks [4]; the methodologies used to test efficacy of these plants [6]; the use of essential oils as deterrents [7]; and a meta-analysis of compounds with activity against ticks [8].

Systematic and standardised determination of in vitro efficacy is a crucial step in the development of botanical acaricides. Farmers often report acaricidal efficacy of some traditional plants against cattle ticks [2, 9, 10] and evidence for this efficacy is required. Laboratory testing helps to determine the effective plant extracts under controlled reproducible conditions. This allows resources for product development to be channelled towards these candidate plant species. One strategy is to start by mimicking farmer practice in the laboratory to determine if farmer observation or experience is replicable in vitro and subsequently in vivo. Most farmers, have only water available as an extractant. It has, however, been established that water alone is not an effective extractant to use for plant-based products because many biologically active compounds are not soluble in water [11].

In a review on research of acaricidal activity of plants, Adenubi et al. [8], listed extractants used by different authors in 392 publications. Acetone was used in 44% of the publications followed by ethyl acetate (20%), petroleum ether (13%), chloroform (12%), methanol (4%), water (4%) and hexane (3%). The low percentage of water used is also an indication that it is not a good extractant for acaricidal compounds. The general efficacy of crude acetone extract for most of the plant species is a clear indication that the active compounds may be of intermediate polarity. Acetone has excellent ability to extract plant-based active compounds in plants and has been used by many researchers [11, 12]. The high percentage of authors using acetone as extractant for acaricidal activity also indicates its low toxicity to ticks [8].

Organic solvents produce better results than water, particularly acetone because of its ability to dissolve cell membranes and extracts both polar and non-polar compounds. It has a low toxicity to target test organisms such as microorganisms [11] and ticks [13]. It has also been shown that solvent-solvent fractionation into different polarity fractions may increase efficacy of plant extracts [14]. Some low-cost measures that can be used include the use of a surfactant and hot water for extraction purposes [15]. In this study, we examined the acaricidal activity of acetone and water extracts with and without a surfactant of different plant species that rural farmers in Zimbabwe use to protect their cattle against ticks. We used *Rhipicephalus (Boophilus) decoloratus*, a very important cattle tick in southern Africa as a test organism in the in vitro assays. We also investigated whether solvent-solvent fractionation could increase the activity of an acetone extract with high activity to facilitate the isolation and characterization of the active compound(s) in the fractions.

The overall objective of the study was to determine the in vitro efficacy of different extractants and fractions of selected plant species against cattle ticks. Based on previous work [15, 16], special attention was given to *M. edulis*.

## Results

### Activity of crude water extracts

When water only was used as an extractant, the extracts had no acaricidal activity with no significant difference with the negative control (distilled water). The addition of a surfactant to water led to an amazing increase in activity compared to the negative control (water with surfactant) in some of the species examined (Table 1). The highest mortality was recorded in the *M. edulis* tuber that was as effective as the amitraz-based positive control (*P* > 0.05). The extracts of *M. lugardae* and *M. edulis* leaves led to mortalities that were below 50% (Table 1). There was no significant difference (*P* > 0.05) between the negative control and the *C. quadrangularis*, *A. vera* and *C. abbreviata* extracts.

**Table 1:** Corrected mortality (%) of tick larvae exposed to crude water and water with surfactant extracts (100 mg/mL) of six plant species and Amitraz (2 mL/L, ; 0.002%) (*N* = 4)

Plant species	Mean mortality (%)	Corrected mortality ±SEM (%)
*Maerua edulis* (tuber)	97.5	97.4 ± 0.96^a^
*Monadenium lugardae* (stems)	32.3	30.6 ± 0.96^b^
*Maerua edulis* (leaves)	15.4	13.3 ± 3.30^c^
*Cissus quadrangularis* (stems)	4.2	1.7 ± 2.34^d^
*Aloe vera* (stems)	5.0	2.6 ± 3.10^d^
*Datura stramonium* (leaves)	19.2	17.5 ± 4.84^c^
*Cassia abbreviata* (leaves)	17.7	15.6 ± 10.16^c^
Water with surfactant (negative control)	2.49	0 ± 0.87^d^
Amitraz (positive control)	100	100^a^
Water without surfactant	0	0^d^

Superscripts with different letters in a column denote treatments that differ statistically (*P* < 0.05)

### Activity of crude acetone extracts

In general, the acetone extracts had higher activity against the tick larvae than water or water with surfactant (Table 2). The most effective acetone extracts were of the *M. edulis* (leaf and tuber) and *Kleinia* sp. (Table 2). There was no statistically significant difference in activity between these treatments and the amitraz-based positive control. The acetone extracts of *M. lugardae* and *C. abbreviata* showed higher activities of 83 and 77% corrected mortalities respectively. With the exception of *A. vera* stems and *C. quadrangularis* stems, all the extracts of the other plants had significantly higher activity than the negative control acetone. The activities of the other plant extracts varied from 20 to 57% corrected mortality (Table 2). There was a high correlation between the activities of the top acetone extracts and the plants extracted using water and a surfactant (*R*^*2*^ = 0.997).

**Table 2:** Corrected mortality (%) of tick larvae caused by acetone extracts (100 mg/mL) of different plant species (*N* = 4)

Plant species	Mean mortality (%)	Corrected mortality ± SEM (%)
*Maerua edulis* (leaf)	97	97 ± 3.3^a^
*Maerua edulis* (tuber)	93	93 ± 6.7^a^
*Kleinia* sp.	90	90 ± 5.8^a^
*Monadenium lurgadae*	83	83 ± 3.3^b^
*Cassia abbreviata*	77	77 ± 6.7^b^
*Cissus quadrangularis*	57	57 ± 6.7^c^
*Aloe excelsa*	53	53 ± 16.7^c^
*Osyris lanceolata*	53	53 ± 12.2^c^
*Albizia amara*	43	43 ± 13.3^d^
*Ricinus communis*	43	43 ± 14.5^d^
*Carissa edulis*	37	37 ± 14.5^d^
*Terminalia sericea*	27	27 ± 12.0^e^
*Croton gratissimus*	23	23 ± 8.8^e^
*Ornithogalum* sp.	20	20 ± 20^e^
Amitraz (positive control)	100	100^a^
Acetone (negative control)	0	0^f^

Corrected mortality values with different superscripts letters are significantly different within the column (*P* < 0.05)

### Solvent-solvent fractionation

The chloroform fractions of the *M. edulis* tuber and leaf and the hexane and butanol fractions of *M. edulis* tuber were the most effective fractions against tick larvae and had no significant difference with the activity of the positive control (amitraz). The other fractions were not as effective (Table 3).

**Table 3:** Corrected mortality (%) of tick larvae treated with solvent- solvent fractions of the acetone extract (10 mg/mL) of *M. edulis* leaves (L) or tubers (T) and Amitraz (0.002% v/v) (*N* = 4)

Plant extract	Mean mortality (%)	Corrected mortality ±SEM (%)
*Maerua edulis* (L) water	32.5	27.2 ± 8.54^c^
*Maerua edulis* (L) butanol	2.0	5.7 ± 0.41
*Maerua edulis* (L) chloroform	100.0	100.0 ± 0^a^
*Maerua edulis* (L) hexane	51.5	47.7 ± 18.53^b^
*Maerua edulis* (T) butanol	96.3	96.0 ± 2.39^a^
*Maerua edulis* (T) chloroform	100.0	100.0 ± 0^a^
*Maerua edulis* (T) hexane	100.0	100.0 ± 0^a^
Amitraz (positive control)	100.0	100.0 ± 0^a^
Diluent (negative control)	7.2	–

Corrected mortality values with different superscript letters within the column are significantly different (*P* < 0.05)

## Discussion

In evaluating the activity of the different extractants, it would have been useful to determine the activity of different concentrations, but this would have meant much additional work. We decided to use a 10% concentration as in our previous publications, because this makes it easy to compare results. Despite being highly rated by farmers [9], no water extracts were effective against the ticks under controlled conditions. Other authors have found that water alone does not extract antimicrobial or pesticidal compounds from plant material because of its high polarity [11, 17]. The most likely compounds to be extracted by water include proteins, sugars and salts which are not toxic to parasites [18]. The possible explanation for the difference may be that rural farmers do not use sterile distilled water. If there are microorganisms in the water that could grow on nutrients present in the plant material, and extraction takes place over a long period, intermediate polarity compounds can be solubilized and kill ticks. Physical factors such as photo-oxidation and temperature may also play a role. It is also possible that some plants contain saponins that act as soap and solubilise intermediate polarity compounds. Finally, the concentrations used by farmers could be higher than the 100 mg/mL used in this study.

It is difficult to discard water as a useful solvent because it is the only extractant generally available and most ethnoveterinary medicines are based on water extracts [19]. The possibility of using water with something added that would solubilize intermediate polarity compounds is logical.

In the solvent-solvent fractionation of the acetone extract of *M. edulis*, the chloroform fraction had the highest activity. This confirms results found in investigating the activity of different extractants and fractions against several microorganisms [14, 20, 21]. It appears that the compounds with intermediate polarity have the highest activity. This could be related to the bioavailability of these compounds to ticks and microorganisms. There was a very large difference in the activity of the n-butanol fraction of leaves and the n-butanol fraction of tubers indicating that different acaricidal compounds may be present in leaves and tubers of *M. edulis.*

It is interesting that there was a very large difference in the activity of *M. edulis* leaves extracted with water and surfactant (15.3% mortality) compared to the acetone extract (97%). This may be related to the difference in using fresh or dried leaves. In the case of the four acetone extracts with the highest activity, there was an excellent correlation between the activities of the water-surfactant and the acetone extracts (*R*^*2*^ = 0.997) confirming that the surfactant succeeded in extracting compounds of the same activity.

## Conclusions

Water, as used under laboratory conditions does not extract acaricidal compounds from any of the 13 plant species used by Zimbabwean smallholder farmers to control ticks according to an ethnoveterinary survey. Acetone has been demonstrated by many authors to be an excellent extractant for antimicrobial, antiparasitic and acaricidal compounds. Acetone extracts of the majority of plants used traditionally to control ticks had good to excellent activity against the cattle tick *R. (B.) decoloratus*. Water is, however, practically the only extractant available for most of the smallholder farmers [18]. Several authors have deduced that intermediate polarity compounds are active against many plant and animal pests. Many plants growing in rural areas contain metabolites with useful activities. By adding 1% of commercial liquid soap, acaricidal compounds were extracted and some of the plant extracts had excellent activity. Adding soap to the water makes it possible for farmers that do not have access to commercial synthetic acaricidal products to exploit the biological activity of pesticidal plants growing in their environment.

## Methods

### Study site

The in vitro studies were conducted in the Phytomedicine Laboratory, Faculty of Veterinary Science, University of Pretoria in South Africa and at the Central Veterinary Laboratories, Harare, Zimbabwe.

### Plant material collection

The plant species collected were in nature from the places around the communities who reported use of them in Muzarabani, Chiredzi, Sanyati and Matopo districts [9]. In Zimbabwe, no special permission is required for collection of plant materials for local research. However, before the survey and subsequent collection of the plant samples, the traditional leadership of the communities were appraised of the intent of the research and the need for collection of samples for further scientific investigations. The plant parts harvested were based on the information received from the farmers in those areas during the ethnobotanical survey [9]. Samples of the plants were taken to the National Herbarium and Botanic Gardens of Zimbabwe for identification and preparation of herbarium voucher specimens. Mr. Christopher Chapano, the resident botanist, identified the plant species and prepared the voucher specimens (Table 4).

**Table 4:** Plant species used and voucher specimen details

Plant species	Family	District collected	Voucher specimen number
*Albizia amara*	Fabaceae	Muzarabani	Nyahangare E38
*Aloe excelsa*	Aloaceae	Muzarabani	Nyahangare E29
*Carissa edulis*	Apocynaceae	Sanyati	Nyahangare E39
*Cassia abbreviata*	Fabaceae	Sanyati	Nyahangare E72
*Cissus quadrangularis*	Vitaceae	Chiredzi	Nyahangare E6
*Croton gratissimus*	Euphorbiaceae	Matopo	Nyahangare E48
*Kleinia* sp.	Asteraceae	Matopo	Nyahangare E50
*Maerua edulis*	Capparaceae	Chiredzi	Nyahangare E5
*Monadenium lurgadae*	Euphorbiaceae	Chiredzi	Nyahangare E15
*Osyris lanceolata*	Santalaceae	Chiredzi	Nyahangare E49
*Ricinus communis*	Euphorbiaceae	Matopo	Nyahangare E42
*Terminalia sericea*	Combretaceae	Matopo	Nyahangare E36
*Ornithogalum* sp*.*	Liliaceae	Chiredzi	Nyahangare E59

### Preparation of plant treatments

#### Preparation of crude water extracts of some species

The plant species selected for water extraction were based on the frequency of use by rural farmers and the absence of well documented investigations on activity. Fresh leaves of *Maerua edulis* Gilg & Ben Dewolf., *Cassia abbreviata* Oliv., *Datura stramonium* L.*, Monadenium lugardae* N.E.Br., fleshy stems of *Cissus quadrangularis* L.*, Aloe excelsa* L. Burm.f. and root tubers of *Maerua edulis* (Table 1) were crushed separately using a pestle and mortar and mixed with distilled water to make 10% *w/v*. A separate set of treatments was prepared where 1% *v/v* surfactant (Sunlight liquid soap produced by Unilever Pty Ltd) was added to optimise the extraction and the extract’s spreading effects before grinding [22]. This liquid soap is widely available in southern Africa. The active components are: sodium dodecylbenzene sulphonate, sodium laurylethersulphonate, sodium xylene sulphonate, ethanol and cocamidopropyl betaine. The percentage of the compounds is a trade secret. The mixtures were left for 24 h after which they were filtered with a Whatman No 1 filter paper to remove the plant residues.

#### Preparation of acetone extracts

A total of 13 plant species used traditionally with different rating by farmers were used: *Monadenium lurgadae* N.E. Br.*, Cassia abbreviate* Oliv.*, Kleinia* sp. Mill., *Maerua edulis* (Gilg. & Benedict) (leaves and tuber), *Cissus quadrangularis* L.*, A. excelsa, Osyris lanceolata* Hochst. & Steud. ex A. DC., *Albizia amara* (Roxb.) Boivin., *Ricinus communis* L., *Carissa edulis* Vahl, *Terminalia sericea* Burch. ex DC., *Croton gratissimus* Burch. var. *gratissimus*, *Ornithogalum* sp. L. The *Kleinia* and *Ornithogallum* plants could not be identified to the species level. This is not too strange in southern Africa containing more than 10% of the world’s species diversity where new species are frequently discovered and the taxonomic treatment of some taxa have not been completed yet. Herbarium voucher specimens are available and the district in which the plant was collected is provided in Table 4 for scientists wanting to repeat the work.

The dried ground plant material (5 g) was mixed with acetone (50 mL), shaken vigorously for 20 min, and then centrifuged at 1700 rpm for 10 min. The supernatant was filtered through a Whatman No. 1 filter paper into pre-weighed glass jars. The extraction procedure was repeated three times for each aliquot of plant material. The solvent was dried under vacuum using a rotary evaporator. After drying, 0.1 g of the residue was dissolved in 1 mL of acetone to make a 10% w/v (or 100 mg/mL) concentration used in the tick bioassays. Previous studies have shown that acetone alone is not toxic or has very low toxicity to tick larvae and other microorganisms and therefore can be used in bioassays [13, 23, 24].

#### Preparation of fractions of acetone extracts of Maerua edulis

The acetone extracts of the leaves and tuber were dried and dissolved in hexane and put in an ultrasonic water bath for 30 min. The solution was transferred to a separating funnel and an equal volume of water added. The water and the hexane fractions were collected separately and the water fraction returned to the separatory funnel. Chloroform was added and an equal amount of distilled water was added. After separation of the fractions, the chloroform and water were collected separately. The water fraction was returned to the funnel and butanol added. After partitioning, the butanol and water fractions were also collected separately. These processes yielded a series of fractions with different polarities. All the extracts were dried under a stream of cold air at room temperature and the dry extracts stored in a cold room at 4 °C. A day before incubation with the tick larvae, approximately 0.2 g of the extract was diluted in approximately 20 mL of double distilled water containing 0.02% Triton X-100 and 1% acetone (diluent). The solution was vortexed for up to 10 min and then put in a sonicator at 37 °C for 10 min to dissolve the extract in the diluent [25]. Undissolved or partially dissolved extracts after these procedures were used without further treatment. The concentrations of the plant extracts (10 mg/mL) were not corrected for incomplete dissolution in the diluent.

### Adapted Shaw Larval Immersion Test (SLIT)

The SLIT method described by Shaw in [25] was used to determine the efficacy of plant extracts against ticks. The method was modified by increasing the larval incubation post treatment to 72 h [18].

### Experimental procedure and data analysis

The procedure described in [25] was followed using 16–21 day old tick larvae in all experiments. Using a soft small paint brush, approximately 200 larvae were placed between two round Whatman No. 1 filter papers (diameter 120 mm) to form a larvae sandwich which was placed in a pie plate (diameter 140 mm). About 10 mL of the test solution from the plant extract was poured carefully over the sandwich to expose the larvae to the plant extract for 30 min. After the 30 min, any excess solution was drained off using paper towels and the sandwich transferred to a clean dry filter paper (diameter 250 mm). The sandwich was opened and each half placed on the dry filter paper. Approximately 100 larvae were brushed off the filter paper to a clean filter paper envelope which was crimped and closed and finally kept in an incubator set at temperature 26 °C ± 2 and relative humidity (RH) 70–90%) [21]. The experiment for each plant extract was duplicated. The 0.1% acetone diluent and Triatix® (12.5% EC amitraz-based compound manufactured by Ecomed Manufacturing, Belmont, Zimbabwe for Coopers Zimbabwe Pty Ltd) applied at the prescribed label dilution rate of 0.002% v/v, were used as the negative and positive controls, respectively. The number of dead larvae was determined after 72 h. The efficacy of each extract was determined by comparing mortality in the test extracts against the mortality in the negative control from which a corrected mortality (CM) was eventually calculated using Abbott’s formula [26]:

$$ \boldsymbol{CM}=\left[\boldsymbol{i}-\frac{\boldsymbol{c}}{\mathbf{100}}-\boldsymbol{c}\right]\ast \mathbf{100} $$

Where **i** = % mortality in test extract; **c** = % mortality in negative solvent control (Diluent); **CM** = % corrected mortality.

## Data Availability

Data is available from the corresponding author.

## Abbreviations

CM: Corrected mortality
Ltd.: Limited
Pvty: Private
RH: Relative humidity
SEM: Standard error of the mean
SLIT: Shaw Larval Immersion Test
